# The Alternative Complement System Mediates Cell Death in Retinal Ischemia Reperfusion Injury

**DOI:** 10.3389/fnmol.2018.00278

**Published:** 2018-08-17

**Authors:** Saori Inafuku, Garrett Klokman, Kip M. Connor

**Affiliations:** ^1^Angiogenesis Laboratory, Massachusetts Eye & Ear Infirmary, Harvard Medical School, Harvard University, Boston, MA, United States; ^2^Department of Ophthalmology, Harvard Medical School, Harvard University, Boston, MA, United States

**Keywords:** retina, complement system, ischemia reperfusion, endothelial cell, shear stress, neurodegeneration

## Abstract

Ischemia reperfusion (IR) injury induces retinal cell death and contributes to visual impairment. Previous studies suggest that the complement cascade plays a key role in IR injury in several systemic diseases. However, the role of the complement pathway in the ischemic retina has not been investigated. The aim of this study is to determine if the alternative complement cascade plays a role in retinal IR injury, and identify which components of the pathway mediate retinal degeneration in response to IR injury. To accomplish this, we utilized the mouse model of retinal IR injury, wherein the intraocular pressure (IOP) is elevated for 45 min, collapsing the retinal blood vessels and inducing retinal ischemia, followed by IOP normalization and subsequent reperfusion. We found that mRNA expression of *complement inhibitors complement receptor 1-related gene/protein-y* (*Crry*), *Cd55* and *Cd59a* was down-regulated after IR. Moreover, genetic deletion of complement component 3 (*C3*^−/−^) and complement factor b (*Fb*^−/−^) decreased IR-induced retinal apoptosis. Because vascular dysfunction is central to IR injury, we also assessed the role of complement in a model of shear stress. In human retinal endothelial cells (HRECs), shear stress up-regulated complement inhibitors *Cd46, Cd55*, and *Cd59*, and suppressed complement-mediated cell death, indicating that a lack of vascular flow, commonly observed in IR injury, allows for complement mediated attack of the retinal vasculature. These results suggested that in retinal IR injury, the alternative complement system is activated by suppression of complement inhibitors, leading to vascular dysfunction and neuronal cell death.

## Introduction

Ischemia reperfusion (IR) injury is a complex pathophysiological phenomenon that is initiated by the loss of blood flow to a tissue and resultant ischemia followed by the subsequent return of blood flow, which results in oxidative stress and downstream cytotoxic inflammatory effects. Retinal ischemia is a major cause of damage in ocular diseases including glaucoma, central retinal vein occlusion and diabetic retinopathy (Osborne et al., 2004; Country, 2017). Current therapeutic approaches for IR injury focus on returning retinal blood flow through the use of anticoagulants, vasodilators, and laser treatment, or by alleviating oxidative stress using free radical scavengers (Osborne et al., 2004). Currently, no clinically available therapeutic modalities are focused on reperfusion-associated inflammatory damage, a major contributor to IR injury. In order to develop these important approaches to treatment, it is essential to identify which inflammatory mediators contribute to retinal IR-induced inflammatory damage.

Ocular immune activity, including activation of the complement cascade, is dramatically increased in multiple forms of ocular IR injury (Ahmed et al., 2004; Kuehn et al., 2006). The complement system is comprised of a complex network of proteins that work together to induce an inflammatory response that signals for removal of non-functional self-cells and non-self-cells. There are three complement pathways (classical, lectin, and alternative) that activate the central component of the complement system, C3, which subsequently activates a final common pathway facilitating cellular clearance (Walport, 2001b; Danese et al., 2007; Ricklin et al., 2010). Evolutionarily, the alternative complement pathway is the oldest of the complement activation pathways, so is broadly conserved (Ricklin et al., 2010). The alternative complement pathway is a key mediator of endogenous immune surveillance and tissue homeostasis. To monitor tissue homeostasis, the alternative complement pathway remains in a constant state of low-level activation, which allows for continuous probing of cells. In contrast, the classical and lectin pathways do not maintain this low-level activation (Pangburn and Müller-Eberhard, 1980; Pangburn et al., 1981; Bexborn et al., 2008; Ricklin et al., 2010).

Activation of the alternative complement pathway occurs through the spontaneous hydrolysis of an internal thioester bond within C3 (Harboe and Mollnes, 2008). Further continuation of the pathway is allowed only by complement factor b (Fb), which binds to C3b deposited in the membrane of a target cell (Walport, 2001a,b). These components form the C3 convertase enzyme, promoting the cleavage of C3 into C3a and C3b, creating a positive feedback activation loop that is down-regulated by complement inhibitors such as Crry/CD46, Cd55, and Cd59 (Hamilton et al., 1990; Rollins and Sims, 1990; Harris et al., 1999).

Soluble and cell-bound regulators of complement help to protect healthy host tissue from self-recognition and serve to prevent activation of a complement response (Song et al., 1996; Song, 2004; Ricklin et al., 2010). However, damaged or diseased host cells may down-regulate membrane bound inhibitors of complement, allowing targeted clearance (Ricklin et al., 2010). An imbalance between complement recognition and initiation on healthy host cells can lead to unregulated complement activation and subsequent cellular damage (Ricklin et al., 2010). Thus, activation of complement not only helps defend the host against pathogens, but also has the potential to affect self-tissues in a both beneficial (protective autoimmunity) and detrimental manner (autoimmunity) (Markiewski and Lambris, 2007; Ricklin et al., 2010).

We previously identified the alternative complement pathway as an important regulator of ocular health in proliferative retinopathy and retinal detachment (Sweigard et al., 2014, 2015; Kim et al., 2016). We found that within the ocular microenvironment, the alternative complement pathway maintains low levels of constitutive activity to ensure the intermittent probing of host cells. In normal conditions, host cells normally express endogenous membrane inhibitors of complement (e.g., *Cd55*) to protect against targeting by the complement system, these inhibitors are down-regulated in response to host cell injury allowing for their targeted removal by the complement system.

In this study, we sought to investigate the role of the alternative complement system in IR injury-induced neuronal damage and vascular dysfunction associated with IR injury. The role of the complement cascade in IR injury has been investigated in many tissues including the kidney (Zhou et al., 2000; de Vries et al., 2003), intestine (Austen et al., 1999; Satyam et al., 2017), lung (Hu et al., 2014), and heart (De Hoog et al., 2014; Chun et al., 2017). These studies suggest complement pathway inhibition as a putative therapeutic approach to alleviate IR-induced cell death and injury. However, the role of the alternative complement pathway in retinal IR injury remains incompletely understood. A previous study identified that activation of the core complement component 3 (C3), was increased following IR injury, and that deletion of C3 alleviated retinal damage and neuron loss after IR injury (Kuehn et al., 2008). This study was important in identifying a role for the complement system in neuronal IR injury, but many elements are as yet unknown, including the role of the alternative complement pathway and how specific compliment inhibitors are regulated in response to IR injury.

Ischemia is categorized by a lack of blood flow to tissue associated with vascular dysfunction (Zhao et al., 2014). Most studies of ischemic disease have focused upon the ramifications of decreased oxygen and nutrient availability. However, blood flow also causes shear stress, which is the frictional force exerted on vessel wall endothelial cells. Physiologic levels of shear stress are important for normal endothelial cell function (dela Paz and D'Amore, 2009). Shear regulates the Kruppel Like Factor 2 (KLF-2) transcription factor, which regulates molecules important to endothelial cell homeostasis including vascular endothelial growth factor (VEGF), endothelial Nitric Oxide Synthase (eNOS), and CD59 (dela Paz et al., 2012). Thus, given the role of vascular dysfunction associated with IR injury and complement's role in targeting injured self-cells we were interested in investigating the role decreased shear stress had on complement inhibitor expression in retinal endothelial cells. The down-regulation of complement inhibitors, occurring as a result of decreased shear stress in IR injury, could contribute to vascular damage and dysfunction through over activation of the complement system.

## Materials and methods

### Animals

All animal procedures adhered to the Association for Research in Vision and Ophthalmology (ARVO) Statement for the Use of Animals in Ophthalmic and Vision Research. The Animal Care and Use Committee of Massachusetts Eye and Ear Infirmary approved the protocol for the experiments outlined herein. C57BL6 mice (stock no. 000664) at 8 week of age were obtained from The Jackson Laboratory (Bar Harbor, ME, USA). *Fb*^−/−^ and *C3*^−/−^ mice were kind gifts of Dr. John Lambris (University of Pennsylvania, Philadelphia, PA, USA). Breeding colonies were maintained at the Massachusetts Eye and Ear Infirmary (MEEI) animal facility. All animals used were between the ages of 8 and 9 weeks for all studies performed.

### Mouse model of retinal ischemia reperfusion (IR)

Mice were anesthetized with Avertin (T4, 840-2; 2,2,2 tribromoethanol; Sigma- Aldrich, St. Louis, MO, USA, in isoamyl alcohol, A730-1; ThermoScientific, Waltham, MA, USA), and deep anesthesia was confirmed by a toe pinch test. Pupils were dilated with 1% tropicamide, and topical anesthesia (1 drop of proparacaine hydrochloride) was applied to cornea. The anterior chamber of the left eye was cannulated with a 30-gauge needle connected to a line infusing sterile saline. The IOP was raised by elevating the saline reservoir to cause ischemia. After 45 min of ischemia, the needle was withdrawn to allow reperfusion. IOP was measured 0, 15, 30, and 45 min after induction of ischemia, before and after the surgery using a TonoLab tonometer (icare, Vantaa, Finland). For sham surgery, a 30-gauge needle was inserted into the anterior chamber without elevating the IOP. Mice were kept on a heating pad until fully awake, as confirmed by upright mobility. Mice were euthanized at various time points after IR, and their retinas were prepared as described below.

### Fundus video and fluorescein angiography

Fundus color video and FA during the IR surgery were taken using the Micron III Retinal Imaging Microscope (Phoenix Research Laboratories, Pleasanton, CA, USA). For FA, 0.1 ml of 2% fluorescein sodium (Akorn, Lake Forest, IL, USA) was injected intraperitoneally. Images were taken by attaching the cornea covered with 2.5% Goniovisc (HUB Pharmaceuticals, Rancho Cucamonga, CA, USA) to the Micron camera lens using StreamPix software (Phoenix Research Laboratories, Pleasanton, CA, USA).

### TUNEL cell death assay

Immediately after enucleation, mouse eyes were placed in optimum cutting temperature (OCT) compound (Tissue Tek, 4583, Torrance, CA, USA), and quickly frozen by submerging in a beaker of isopropanol chilled by dry ice. All eyes were cut into 10 μm sections with four sections per eye.

TUNEL was performed using the ApopTag Fluorescein *In Situ* Apoptosis Kit (S7110; Millipore, Billerica, MA, USA) following the manufacturers instructions. The sections were coverslipped with 4′,6-diamidino-2-phenylindole (DAPI) containing medium (H-1200; Vector Laboratories, CA, USA). All images were obtained using an AxioVision microscope (Zeiss, Chester, VA, USA), and the TUNEL Cell Counter plugin in Fiji image analysis software was used to automatically calculate the area and number of TUNEL-positive cells. When using the TUNEL Cell Counter plugin, the retina area was selected manually and the threshold sensitivity was changed to high in all the images, while all other settings remained unchanged. Eight images were taken in the midperiphery of each retina using a 20X objective lens.

### Hematoxylin and eosin (H & E) staining

Mice were euthanized on day 7 following IR or sham surgery, and eyes were enucleated. All eyes were fixed in 4% paraformaldehyde and paraffin embedded. Sections (6-μm thick) were cut parallel to the maximal circumference of the eye ball through the optic nerve and stained with hematoxylin and eosin (H&E). Inner nuclear layer (INL) thickness was measured in eight areas within 200–500 μm from the optic nerve, and the mean value was calculated.

### Cell culture

Human retinal endothelial cells (HRECs) were purchased from Cell Sytems, Inc. (ACBRI 181; Kirkland, WA, USA) and grown in EGM-2 Growth Medium with SingleQuots (Bulletkit CC-3162; Lonza, Basel, Switzerland) supplemented with 1% L-glutamine and penicillin-streptomycin. They were grown to 90% confluence in T75s coated with 0.2% gelatin under the following incubator conditions: 5% CO_2_, 37°C, and 95% humidity. HRECs used for experiments were from passage 7–9.

### Shear stress model

Cells were seeded into three wells of a six-well plate at a density of 50,000 cells/well. For each experiment, one plate was used for each magnitude of shear stress. Cells were synchronized by culturing in starvation medium [EBM-2 basal medium (CC-3156; Lonza), 5% calf serum, 25 mM N-2-hydroxyethylpiperazine-N′-2-ethanesulfonic acid (HEPES), L-glutamine, and penicillin-streptomycin] for 24 h. After 24 h, fresh starvation medium was added and plates were exposed to orbital shear stress for 24 h using orbital shakers (Orbi-Shaker JR BT300; Benchmark Scientific, Sayreville, NJ, USA) set to either ~5 (150 rpm) or ~10 (240 rpm) dynes/cm^2^ inside an incubator. Cells of the same passage were placed in the same incubator as the static control. Shear stress was approximated using the formula ${\text{T}}_{\text{max}}=\alpha \sqrt{{\rho \eta \left(2\pi f\right)}^{3}}$, where α is the radius of orbital rotation (0.95 cm), ρ is the density of the medium (1.0 g/ml), η is the viscosity of the medium poise (7.5 × 10^−3^ dynes·s/cm^2^), and f is the frequency of rotation (rotations/second) (Dardik et al., 2005; dela Paz et al., 2012).

### LIVE/DEAD assay

HRECs were cultured in starvation medium supplemented with 1% mouse serum, while being exposed to shear stress (0, 5, or 10 dynes/cm^2^) for 24 h. The percentage of dead cells was determined using the ReadyProbes Cell Viability Imaging Kit (ThermoFisher Scientific, Waltham, MA, USA). Three representative images of the peripheral area were taken per well using Zeiss Zen imaging software. Without any post processing, Fiji image analysis software was used to automatically count the number of dead cells (green) and total cells (blue) using the particle analyzer tool. The number of dead cells and total cells of the three images taken for each well were averaged to calculate the percent cell death per well.

### RNA isolation and cDNA preparation

For retinal gene expression analysis, eyes were collected 6 h after IR surgery or sham surgery, and dissected retinas were flash frozen in liquid nitrogen. For HRECs, cells were treated with trypsin, pelleted by centrifugation, and flash frozen. RNA was collected using the RNeasy Mini Kit (Qiagen, Hilden, Germany) following the manufacturers instructions. Total RNA was measured using the NanoDrop 2000 Spectrophotometer (ThermoFisher Scientific). cDNA was normalized prior to reverse transcription with SuperScript III and random hexamer primers (Invitrogen, Carlsbad, CA, USA).

### Real-time PCR analysis

RT-PCR was performed using 1 μl of cDNA and a StepOnePlus Real-Time PCR System (Applied Biosystems, Waltham, MA, USA). Primers to *Crry* (Mm00787529_s1, ThermoFischer Scientific), *Cd55* (Mm00438377_m1, ThermoFischer Scientific), *Cd59a* (Mm00483149_m1, ThermoFischer Scientific) were used for mouse retinas, and primers to *Cd46* (Hs00611257_m1, ThermoFischer Scientific), *Cd55* (Hs00892618_m1, ThermoFischer Scientific), and *Cd59* (Hs00174141_m1; ThermoFischer Scientific) were used for HRECs with TaqMan Universal PCR Master Mix (4304437, ThermoFischer Scientific). All data was normalized to β-actin.

### Statistical analysis

Data were analyzed using one-way ANOVA. Results are expressed as mean ± SEM. Significance was set at *P* < 0.05.

## Results

### Circulation was obstructed in the IR model

For the retinal IR model, we applied a widely-used method, which causes retinal ischemia by increasing the intraocular pressure (IOP) above the ocular perfusion pressure (Zhi et al., 2012, 2015; He et al., 2013). In this model, the anterior chamber, located directly behind the cornea, is cannulated with a needle connected to a sterile saline reservoir to increase intraocular pressure and collapse retinal blood vessels, inducing retinal ischemia. The blood supply deficiency results in oxygen and nutrient deprivation of the retina, causing retinal cell death.

**Figure 1:**
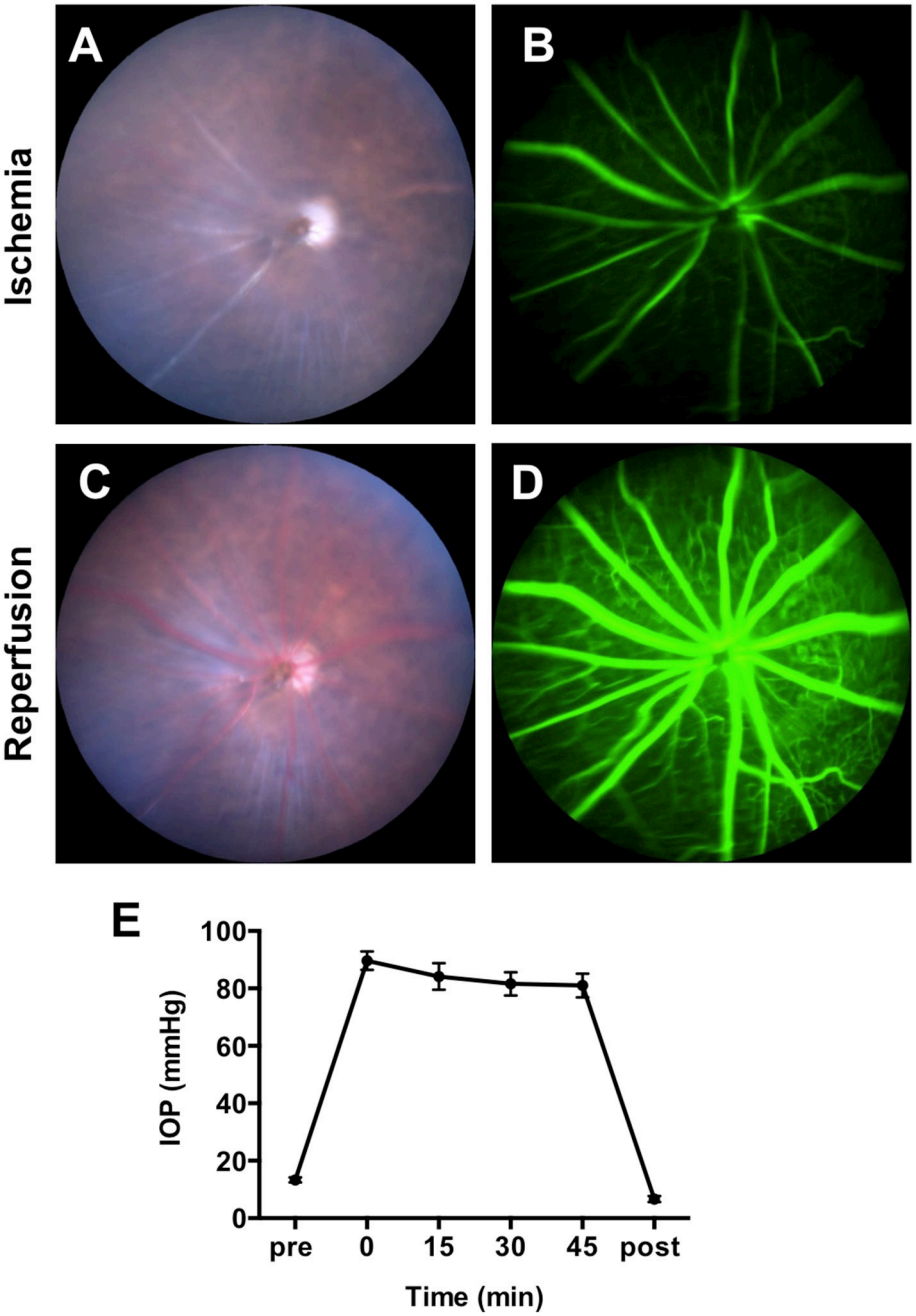
Obstruction of blood flow and the intraocular pressure (IOP) changes during IR surgery. Representative images of fundus color video **(A)** and fluorescein angiography **(B)** during the ischemia phase of IR surgery. Representative images of fundus color video **(C)** and fluorescein angiography **(D)** during the reperfusion phase of IR surgery. **(E)** IOP was measured before the IR surgery, 0, 15, 30, and 45 min after the initiation of the ischemia phase and immediately after the reperfusion. *N* = 12 eyes/time point. Data are presented as the mean ± SEM.

To confirm the obstruction of the blood flow by IR surgery, we recorded color fundus videos and fluorescein angiography (FA) videos during the IR operation (Supplementary Video 1). Immediately after the elevation of the IOP, constriction of vessels and whitening of the whole retina were observed in the color fundus video (Figure 1A and Supplementary Video 1). Utilizing FA, remarkable slowing of blood flow and a lack of choroidal filling were observed (Figure 1B and Supplementary Video 2). When the needle was removed from the anterior chamber, blood flowed in from the central retinal artery instantaneously, allowing retinal vessels to revert to their normal width and the retina to regain its original color (Figure 1C and Supplementary Video 3). FA demonstrated that blood flow reverted to a normal velocity, and that choroidal filling was restored (Figure 1D and Supplementary Video 4). We next assessed IOP during the IR operation. IOP was measured before the IR surgery, 0, 15, 30, and 45 min after the onset of IR, and immediately after the removal of the needle. Mean IOP before the surgery (13 ± 0.3 mmHg) was within normal range. By the onset of the IR, mean IOP increased to 90 ± 1 mmHg and remained over 80 mmHg during the surgery (84 ± 1mmHg at 15 min, 82 ± 1 mmHg at 30 min, and 81 ± 1 mmHg at 45 min) (Figure 1E). IOP decreased immediately upon removal of the needle from the anterior chamber. There was no significant difference in IOP between male and female animals at all time points (Supplementary Images 1A,B). Taken together, these results indicate that the IR model impaired blood supply from both choroidal and retinal circulation, and that ischemia was maintained during the 45-min surgery.

### Inner nuclear layer apoptosis was maximal at 24 h after reperfusion

**Figure 2:**
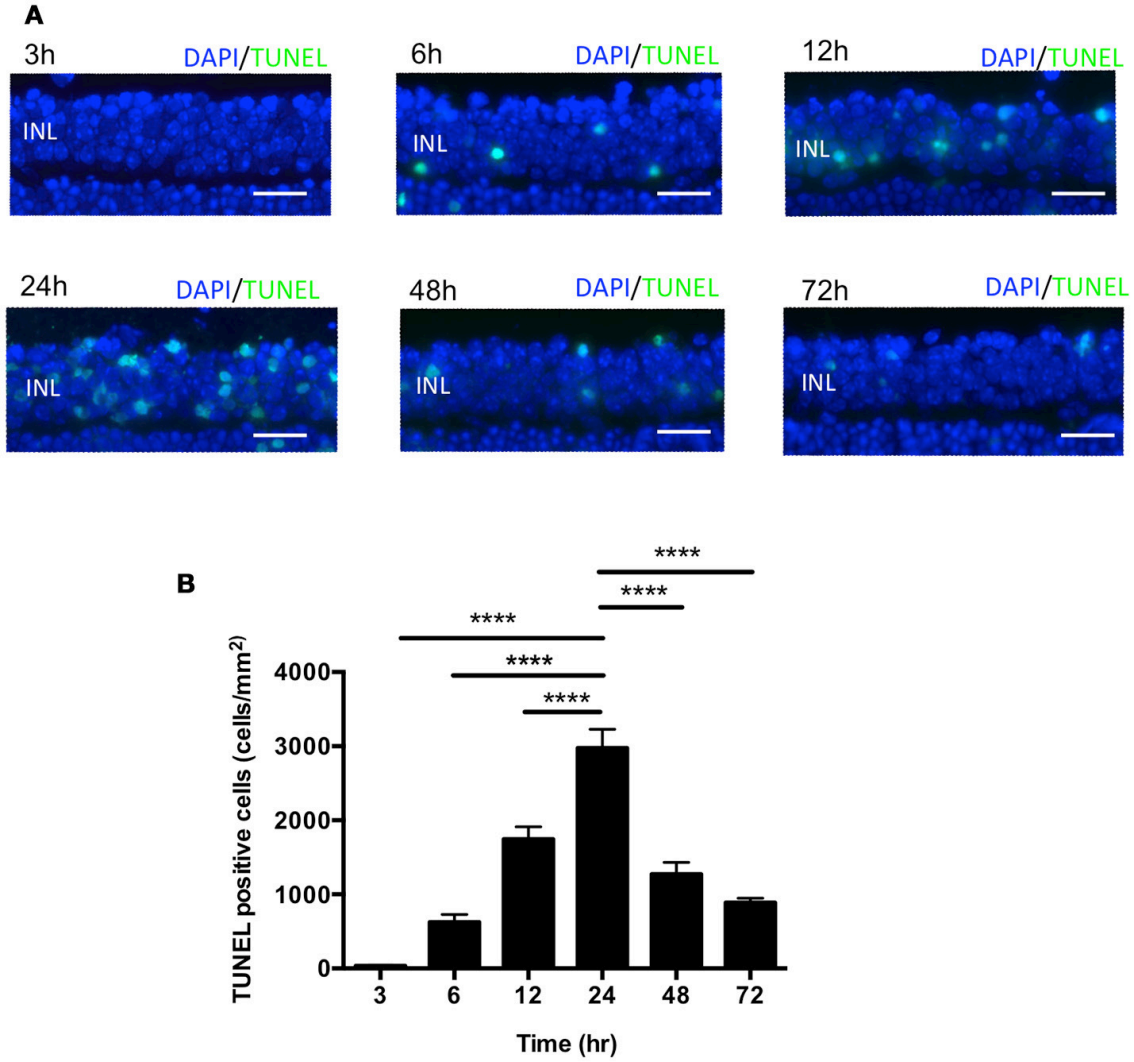
IR induced apoptosis peaked 24 h after reperfusion. **(A)** Representative images of TUNEL labeling of retinal cross-sections at 3, 6, 12, 24, 48, and 72 h after IR injury co-labeled with DAPI (blue) and TUNEL (green). Scale bars = 20 μm. INL, inner nuclear layer. **(B)** Quantification of TUNEL-positive cells in the INL at 3, 6, 12, 24, 48, and 72 h after IR injury. *N* = 8–10 eyes/time point. Data are presented as the mean ± SEM. ^****^P < 0.0001 vs. 24 h.

In the mouse model of IR injury the inner nuclear layer (INL) of the retina is more prone to reproducible irreversible cell loss caused by IR injury, thus we sought to assess cell death in the INL for our studies (Hughes, 1991). To assess changes in INL cell death following acute ocular hypertension, we performed terminal deoxynucleotidyl transferase dUTP nick end labeling (TUNEL) staining of retinas at 3, 6, 12, 24, 48, and 72 h after reperfusion. The number of apoptotic cells peaked 24 h after reperfusion (2976 ± 252 cells/mm^2^), significantly higher than other time points (Figures 2A,B). There was no difference in INL TUNEL positivity between male and female mice at all the time points (Supplementary Image 2). Based on this result, we analyzed cell death 24 h after reperfusion in subsequent mechanistic studies.

### Retinal expression of complement inhibitors is down-regulated in response to IR

**Figure 3:**
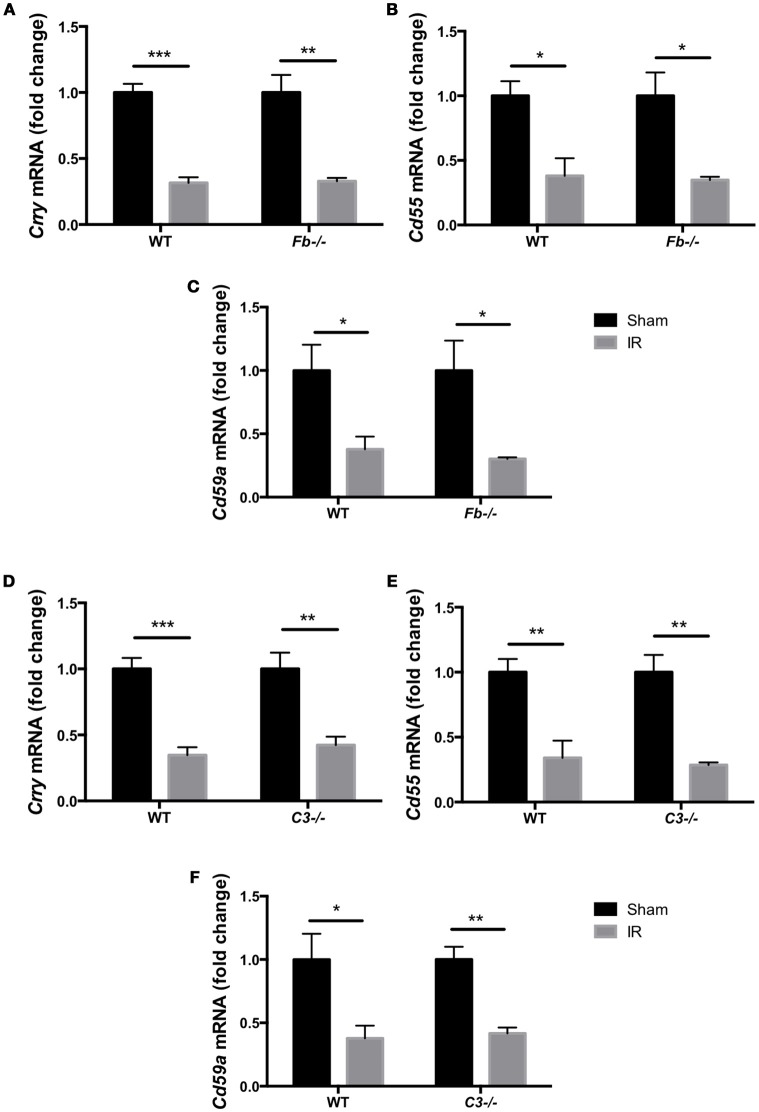
Complement inhibitors gene expression was down-regulated in the IR retina. The mRNA levels of complement inhibitors, *Crry* **(A,D)**, *Cd55* **(B,E)**, and *Cd59a* **(C,F)** in the retina of wild type control mice (WT), complement factor b-deficient mice (*Fb*^−/−^) and complement component 3-deficient mice (*C3*^−/−^) at 6 h after IR injury were quantified by RT-PCR, and were normalized to sham surgery. *N* = 4 eyes/group. Data are presented as the mean ± SEM. *P < 0.05 vs. sham, ^**^P < 0.01 vs. sham, ^***^P < 0.001 vs. sham.

The complement system acts as an immune surveillance system to discriminate among healthy host tissue, cellular debris, apoptotic cells, varying its response in reaction to cellular injury (Ricklin et al., 2010). Generally, normal healthy host cells endogenously express membrane-bound complement inhibitors to prevent self-recognition and targeting by the complement system, thereby protecting themselves from complement-mediated cell death (Powers et al., 2006; Swirski et al., 2009; Rice et al., 2010). However, damaged host cells down-regulate complement inhibitors, allowing for targeted clearance of damaged cells (Powers et al., 2006; Pacher and Szabo, 2008; Bamboat et al., 2010). To investigate whether the complement pathway is involved in IR injury-induced retinal cell death, we evaluated the expression of complement inhibitors *complement receptor 1-related gene/protein-y* (*Crry*), *Cd55* and *Cd59a* in C57BL6 mice, *Fb*^−/−^ mice and *C3*^−/−^ mice at 6 h after IR surgery or sham surgery. The complement inhibitors were significantly decreased in the retina after IR surgery in all three strains (Figures 3A–F). This result suggests that the complement cascade may be activated by suppression of complement inhibitors in IR injury.

### Retinal cell death was suppressed in complement pathway-deficient mice

The complement system is comprised of three pathways (classical, lectin, and alternative pathways). All three pathways activate the central complement component 3 (C3). Subsequent entry into a final terminal pathway results in membrane attack complex (MAC) formation, which creates a pore in the cell membrane and leads to cell lysis and death (Walport, 2001a; Danese et al., 2007; Ricklin et al., 2010).

**Figure 4:**
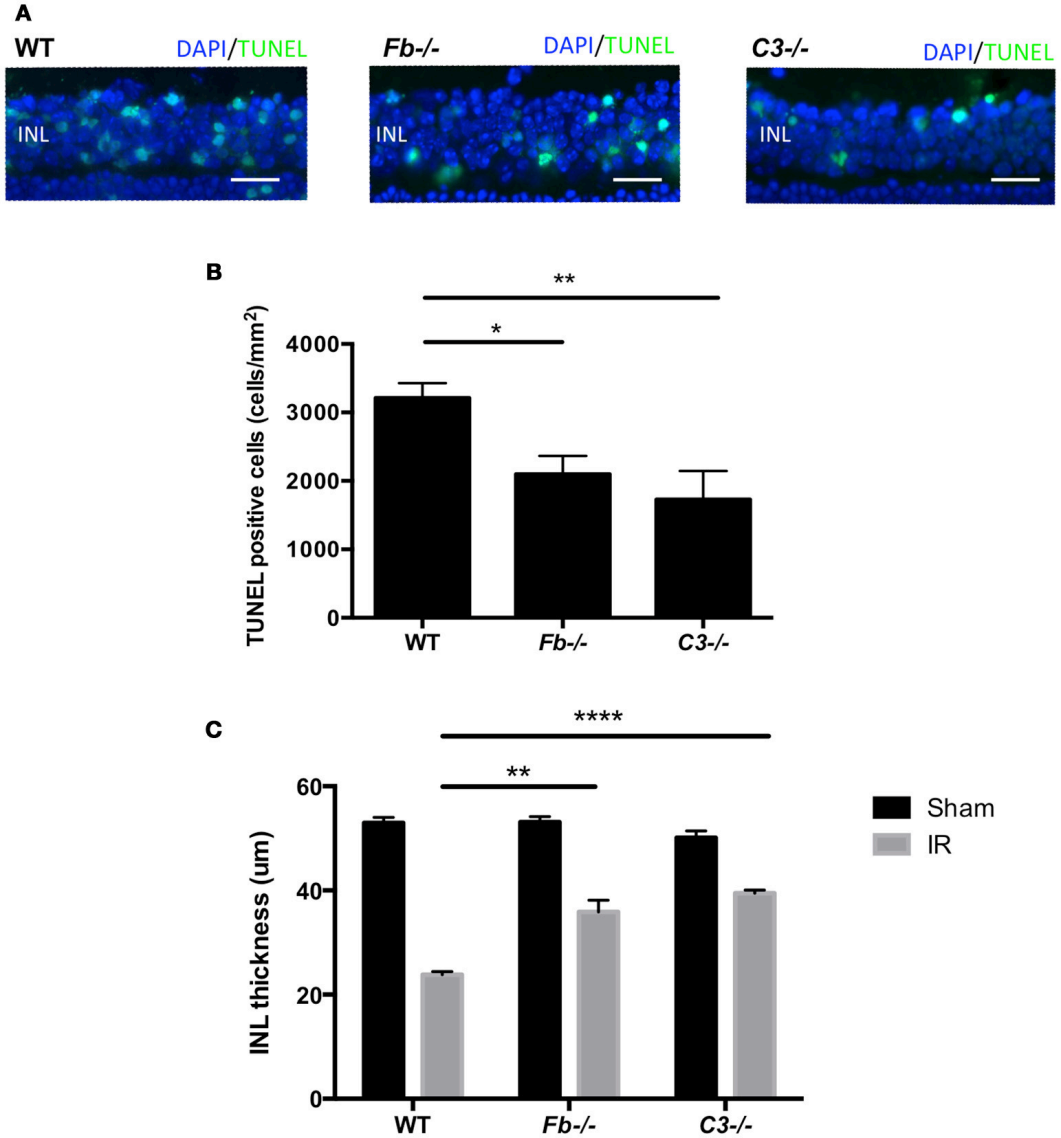
IR induced retinal cell death and degeneration is suppressed in complement-deficient mice. **(A)** Representative images of TUNEL labeling of retinal cross-sections at 24 h after IR injury in wild type control mice (WT), complement factor b-deficient mice (*Fb*^−/−^) and complement component 3-deficient mice (*C3*^−/−^), colabeled with DAPI (blue) and TUNEL (green). Scale bars = 20μm. INL, inner nuclear layer. **(B)** Quantification of TUNEL-positive cells in the INL of WT control, *Fb*^−/−^ and *C3*^−/−^ mice at 24 h after IR injury. *N* = 11–14 eyes/group. Data are presented as the mean ± SEM. *P < 0.05 vs. WT, **P < 0.01 vs. WT. **(C)** The thickness of the INL was measured 7 days after IR injury or sham surgery. *N* = 6 eyes/group. Data are presented as the mean ± SEM. ^**^P < 0.01 vs. WT, ^****^P < 0.0001 vs. WT.

To elucidate the role of the complement system in retinal cell death caused by IR, we performed retinal TUNEL staining in IR-injured *C3*^−/−^ mice relative to wild-type controls. *C3*^−/−^ mice lack the central C3 protein required for all three complement pathways. Cell death was quantified at 24 h after IR, the peak of cell death. The number of TUNEL-positive cells in *C3*^−/−^ mice (1727 ± 418 cells/mm^2^) decreased by 46% compared to C57BL6 mice (3213 ± 216 cells/mm^2^) (Figures 4A,B).

To further elucidate the role (Geijer and Bill, 1979) of the complement system in retinal IR injury, we next measured retinal cell death in complement factor b-deficient (*Fb*^−/−^) mice, which are deficient in the effector of the alternative pathway. The number of apoptotic cells in *Fb*^−/−^ mice (2098 ± 268 cells/mm^2^) was down-regulated by 35% compared to C57BL6 mice (Figures 4A,B). There was no difference in INL TUNEL positivity between male and female mice of both strains (Supplementary Image 3).

In order to evaluate the long-term effect of IR injury, we analyzed INL thickness in C57BL6 mice, *Fb*^−/−^ mice and *C3*^−/−^ mice 7 days after IR surgery or sham surgery. When compared to C57BL6 mice (24 ± 1 μm), a reduction of INL thickness after IR injury was suppressed by 47% in *Fb*^−/−^ (38 ± 2 μm) and by 63% in *C3*^−/−^ mice (40 ± 1 μm). This demonstrated that IR injury induces morphologic changes in the retina, and revealed the long-term protective effect conferred by the lack of C3 or Fb (Figure 4C). These results indicate that the alternative complement pathway contributes to the retinal cell death induced by IR injury.

### Shear stress has a protective effect against complement mediated cell death

The main factor in IR injury pathology is the loss of blood flow, leading to vascular dysfunction as well as neuronal damage. Most investigations of ischemic disease have focused on the consequences of resultant oxygen and nutrient deprivation. However, loss of blood flow also results in loss of shear stress in endothelial cells lining the vessel walls, which is important for normal endothelial cell homeostasis (Ballermann et al., 1998). Therefore, we hypothesized that shear stress may also play a role in IR injury. Endothelial cells are known producers of complement, and we sought to investigate whether shear stress affected complement activation in endothelial cells.

**Figure 5:**
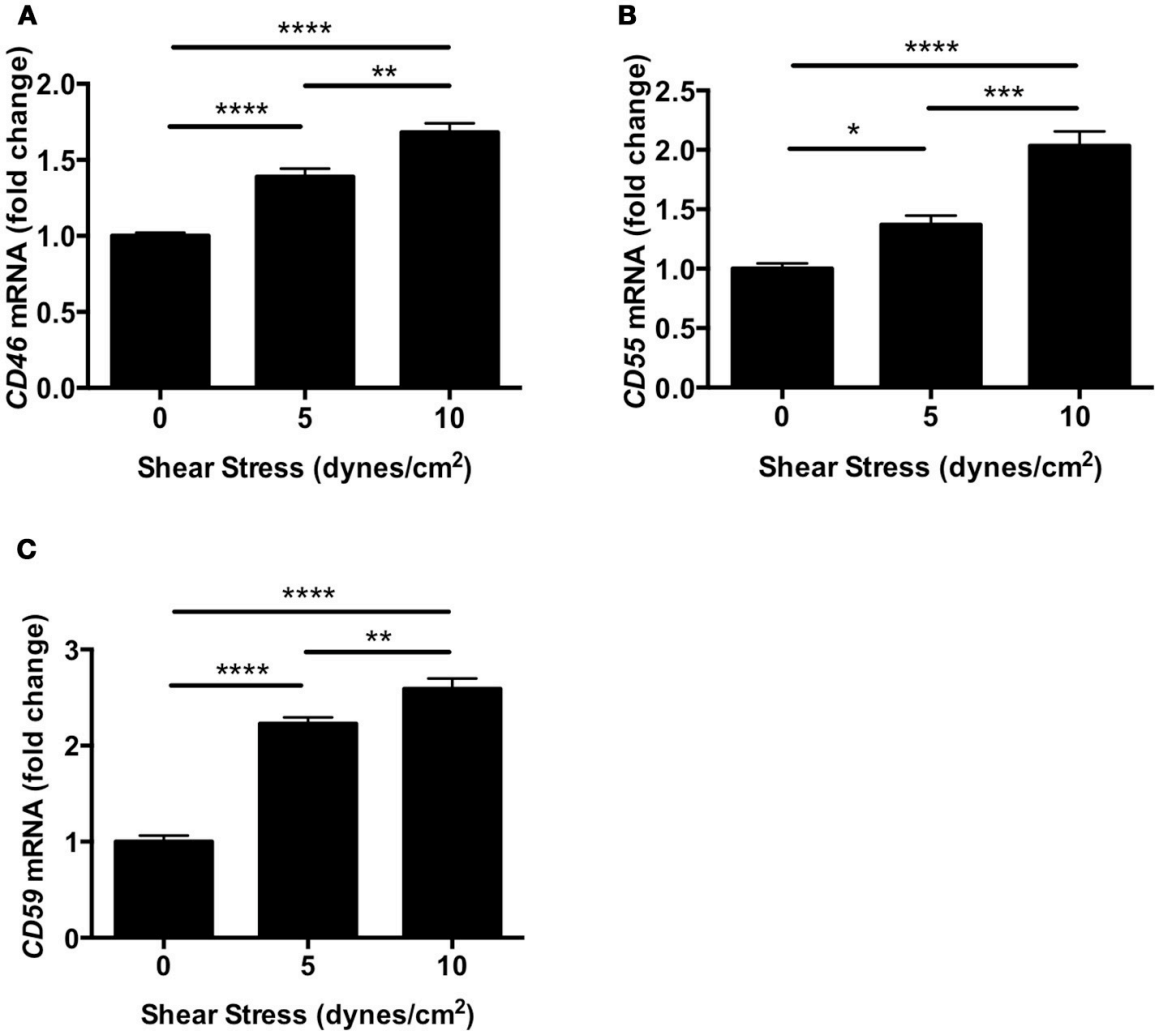
Complement inhibitors were up-regulated in HRECs under shear stress. **(A–C)** The mRNA levels of complement inhibitors, *CD46* **(A)**, *Cd55* **(B)**, and *Cd59* **(C)** in HRECs were quantified by RT-PCR after exposure to orbital shear stress (5 or 10 dynes/cm^2^) for 24 h, and were normalized to control (0 dynes/cm^2^). The expression of complement inhibitors was increased in proportion to the magnitude of the shear stress applied. Data are the average of four independent experiments. Data are presented as mean ± SEM. ^*^P < 0.05, ^**^P < 0.01, ^***^P < 0.001, ^****^P < 0.0001.

To investigate the effect of shear stress on complement inhibitors, we evaluated the expression of *Cd46, Cd55*, and *Cd59* in human retinal endothelial cells (HRECs). The expression of complement inhibitors was determined by real-time polymerase chain reaction (RT-PCR) after exposure to orbital shear stress (0, 5, or 10 dynes/cm^2^) for 24 h. All three complement inhibitors were significantly up-regulated by shear stress in proportion to the relative amount of shear stress applied (Figures 5A–C).

**Figure 6:**
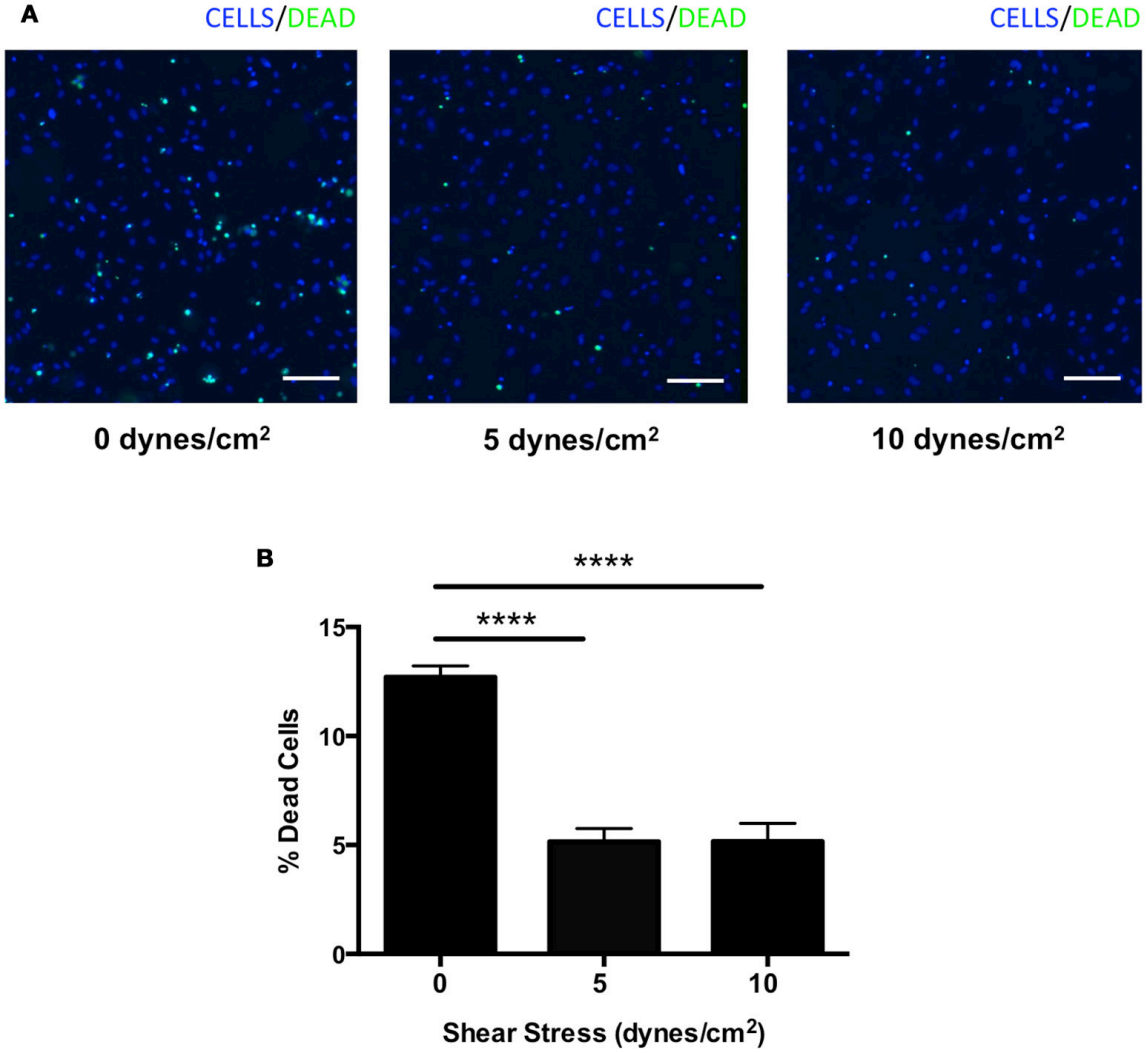
Complement mediated cell death was suppressed in HRECs by shear stress. **(A)** Representative images of HRECs stained with a cell viability imaging kit after exposure to orbital shear stress (0, 5, or 10 dynes/cm^2^) for 24 h during incubation in media with foreign complement. Cells (blue), dead cells (green). Scale bars = 100 μm. **(B)** The percentage of dead cells in HRECs after exposure to to orbital shear stress (0, 5, or 10 dynes/cm^2^) for 24 h with media containing foreign complement. The cell death was significantly suppressed in HRECs exposed to shear stress. Data are the average of five independent experiments. Data are presented as the mean ± SEM. ^****^P < 0.0001 vs. 0 dynes/cm^2^.

We next assessed whether shear stress may protect against complement-mediated cell death. HRECs were exposed to shear stress (0, 5, or 10 dynes/cm^2^) for 24 h during incubation in media containing 1% mouse serum as a source of foreign complement. Cells were stained with a viability imaging kit, and the percentage of dead cells was calculated. The percentage of HRECs undergoing cell death was significantly suppressed by 60% in both 5 and 10 dynes/cm^2^ magnitudes (5 ± 1 and 5 ± 1%, respectively) compared to 0 dynes/cm^2^ magnitude (13 ± 1%) (Figures 6A,B). These data indicate that shear stress may contribute to protection from complement-mediated cell death by up-regulating expression of complement inhibitors.

## Discussion

IR injury is a well-established model of retinal vascular occlusion diseases such as acute glaucoma and diabetic retinopathy (Büchi et al., 1991). Although the elevation of IOP is well-known to affect retinal perfusion (Geijer and Bill, 1979; Zhi et al., 2012, 2015; He et al., 2013), the IOP during the ischemic phase of IR surgery ranges from 60 to 120 mmHg, and the reservoir height used is not standardized, meaning that there may be significant variation between investigators (Zheng et al., 2007; Huang et al., 2013; Hu et al., 2015; Seong et al., 2017; Zanoni et al., 2017). Furthermore, ischemia and reperfusion are usually confirmed indirectly by observation of the iris, episcleral veins or the reflex from the fundus (Da and Verkman, 2004; Oharazawa et al., 2010; Yokota et al., 2011; Xiong et al., 2017). Few studies have focused on observing the ischemia and reperfusion directly, meaning that the degree of ischemia and reperfusion attained could vary between groups. In this study, we demonstrated that blood supply from both the choroidal and retinal circulation is obstructed immediately by elevating the IOP to 80 mmHg, and reperfused by the removal of the needle (Supplementary Videos 1A–D).

While several studies have reported a time course of apoptosis in the IR model, the results between investigators were variable. In our IR model, TUNEL-positive cells in INL were found as early as 3 h after the reperfusion, peaked at 24 h, and decreased gradually over subsequent time points. This result is consistent with a previous report evaluating apoptotic cells in the INL of IR-injured rat retinas (Hu et al., 2015). The earliest evaluated time point in most prior studies was 6 or 12 h post-reperfusion. We also measured retinal apoptosis 3 h post-reperfusion, and found that apoptosis began to occur earlier than previously reported (Huang et al., 2013; Hu et al., 2015).

The complement system is known to be a crucial mediator of systemic IR injury (Austen et al., 1999; de Vries et al., 2003). However, little is known about complement-mediated cell death in retinal IR injury. In the present study, we demonstrated that compliment inhibitors *Crry, Cd55*, and *Cd59a* were suppressed early on in the retina (6 h) after IR injury in C57BL6 mice, *Fb*^−/−^ mice and *C3*^−/−^ mice. These inhibitors are regarded as critical negative regulators of the complement system due to their ability to modulate all three complement pathways. Crry inhibits C3 convertase (Harris et al., 1999), CD55 inhibits both C3 convertase and C5 convertase, and CD59 inhibits formation of the MAC (Hamilton et al., 1990; Rollins and Sims, 1990). These results are consistent with our previous study reporting that photoreceptors are susceptible to targeted cell death due to down-regulation of complement inhibitors (Sweigard et al., 2015). Furthermore, in the mouse renal IR model, CD55-deficient and CD55/CD59-double deficient mice were found to develop more severe IR injury and tubular damage, accompanied by endothelial deposition of C3 and the MAC (Yamada et al., 2004). Our findings indicate that down-regulation of complement inhibitor expression increases activation of the complement pathway, and eventual death of retinal cells.

The complement cascade plays a well-established role in systemic IR injury (Zhou et al., 2000; Orsini et al., 2016; Chun et al., 2017; Satyam et al., 2017). Several studies have reported decreased IR injury in mice deficient in the complement system. In *C3*^−/−^ mice, which lack the central C3 protein required for all three complement pathways, renal tubule damage (Zhou et al., 2000), intestinal damage (Satyam et al., 2017), optic nerve and retinal ganglion cells damage (Kuehn et al., 2008) after IR injury was decreased relative to wild-type controls. In *Fb*^−/−^ mice, which are deficient in an essential rate-limiting protein for alternative pathway activation (Nielsen et al., 1992), decreased myocardial necrosis (Chun et al., 2017) and neurological deficits (Orsini et al., 2016) were observed after IR injury. In our retinal IR model, cell death in the INL was suppressed in *C3*^−/−^ mice compared to wild-type (WT) mice. Furthermore, a similar reduction in the amount of retinal cell death was observed in *Fb*^−/−^ mice. Consistent with these results, when the thickness of the INL was measured 7 days following IR injury in WT, *C3*^−/−^ and *Fb*^−/−^ mice, complement deficient strains were found to have significant protection against INL degeneration compared to WT mice in response to IR injury (Figure 4C). As factor B is exclusive to the alternative pathway, these results suggest that the complement system, especially the alternative pathway, likely contributes to IR injury-induced retinal cell death. We previously found that the classical and lectin pathways play a minor role in a oxygen-induced retinopathy model and photoreceptor cell death retinal detachment model, using classical pathway knockout mice (*C1q*^−/−^) and lectin pathway knockout mice (*Mbl A/C*^−/−^) (Sweigard et al., 2014, 2015; Kim et al., 2016). Thus, it would be interesting to assess the contribution of these pathways in this IR model in a future study.

Our current *in vitro* experiments showed that apoptotic cell death was suppressed by shear stress in HRECs. This result was consistent with previous studies demonstrating that shear stress protects human umbilical vein endothelial cells (HUVECs) from apoptosis (Dimmeler et al., 1996; dela Paz et al., 2012). Furthermore, we also demonstrated that complement inhibitors *Cd46, Cd55*, and *Cd59* are up-regulated by shear stress. However, earlier work has illustrated that *Cd59* expression was increased by shear stress, but no significant difference in *Cd46* and *Cd55* expression was observed in HUVECs (Kinderlerer et al., 2008). One possible explanation for this is the difference in model of shear stress. While the previous study group used a parallel plate flow chamber, which induces laminar shear stress, we used the orbital plate method, which induces orbital shear stress. Prior studies suggest that orbital shear stress more closely mimics disturbed or turbulent flow, and causes an increase in endothelial cell proliferation and apoptosis compared to laminar shear stress (Dardik et al., 2005). This is due to the uniformity of the shear stress in the plate. The peripheral cells are exposed to more shear stress compared to the cells in the center (Dardik et al., 2005). Endothelial cells are known producers of complement (Fischetti and Tedesco, 2006), and it is possible that the vasculature stops producing complement inhibitors in the absence of shear stress. Identifying TUNEL positivity in vessels in retinal cross-sections after IR has proven to be technically difficult, likely due to the small cross-sectional area of these structures. However, it is likely that in IR injury, like stroke, vessels become dysfunctional and injured, but are by-in-large protected from the temporary ischemic conditions. Supporting this was the finding that interruption of shear stress for 45 min did not cause significant difference *in vitro*. This is likely due to differences between an *in vivo* system and a cell culture system. The cells in culture are likely able to adapt more rapidly due to their immortalized nature and normal growing conditions of 20% oxygen.

In conclusion, we found that complement system plays a key role in IR injury-induced retinal cell death. Our data suggest that the lack of blood flow may contribute to the suppression of complement inhibitors, leading to apoptotic cell death via activation of the complement system.

## Data availability

All datasets for this study are included in the manuscript and the supplementary files.

## Author contributions

SI, GK, and KC designed the study. SI and GK conducted experiments, and analyzed data. SI and KC wrote the manuscript. All authors have been involved in drafting the manuscript critically for important intellectual content.

### Conflict of interest statement

The authors declare that the research was conducted in the absence of any commercial or financial relationships that could be construed as a potential conflict of interest.

## Supplementary material

Supplementary Image 1 No gender difference was observed in intraocular pressure (IOP) during ischemia reperfusion (IR) surgery. **(A,B)** Mean IOP before, after and during the 45-min IR surgery in male **(A)** and female **(B)** mice. *n* = 6 eyes/time point. Data are presented as the mean ± SEM.

Supplementary Image 2 No gender difference was observed in time course of ischemia reperfusion (IR) induced apoptosis. Quantification of TUNEL-positive cells in the INL at the indicated time points after IR. TUNEL, terminal deoxynucleotidyl dUTP nick end labeling; M, male; F, female. *n* = 4–5 eyes/time point. Data are presented as the mean ± SEM. N.S., not significant.

Supplementary Image 3 No gender difference was observed in ischemia reperfusion (IR) induced apoptosis in complement pathway-deficient mice. Quantification of TUNEL-positive cells in the INL in WT control, *Fb*^−/−^ and *C3*^−/−^ mice after IR. TUNEL, terminal deoxynucleotidyl dUTP nick end labeling; WT, wild type control. *n* = 5–8 eyes/group. Data are presented as the mean ± SEM. N.S., not significant.

Supplementary Videos 1–4 Color fundus videos **(1)** and **(3)** and fluorescein angiography (FA) videos **(2)** and **(4)** illustrating vascular collapse and reperfusion during the IR model. Color fundus **(1)** and FA **(2)** videos converting from the normal perfusion phase to the ischemic phase. Color fundus **(3)** and FA **(4)** videos converting from the ischemic phase to the reperfusion phase.
